# Desmoglein 2 and desmocollin 2 depletions promote malignancy through distinct mechanisms in triple-negative and luminal breast cancer

**DOI:** 10.1186/s12885-024-12229-2

**Published:** 2024-04-26

**Authors:** Ji-Yuan Han, Na Che, Jing Mo, Dan-Fang Zhang, Xiao-Hui Liang, Xue-Yi Dong, Xiu-Lan Zhao, Bao-Cun Sun

**Affiliations:** 1 Department of Pathology, School of Basic Medical Science, Tianjin Medical University, 300070 Tianjin, China; 2 Department of Pathology, General Hospital of Tianjin Medical University, 300052 Tianjin, China

**Keywords:** Desmoglein 2 (Dsg2), Desmocollin 2 (Dsc2), Breast cancer, ERK, AKT, β-catenin

## Abstract

**Background:**

Aberrant expressions of desmoglein 2 (Dsg2) and desmocollin 2(Dsc2), the two most widely distributed desmosomal cadherins, have been found to play various roles in cancer in a context-dependent manner. Their specific roles on breast cancer (BC) and the potential mechanisms remain unclear.

**Methods:**

The expressions of Dsg2 and Dsc2 in human BC tissues and cell lines were assessed by using bioinformatics analysis, immunohistochemistry and western blotting assays. Wound-healing and Transwell assays were performed to evaluate the cells’ migration and invasion abilities. Plate colony-forming and MTT assays were used to examine the cells’ capacity of proliferation. Mechanically, Dsg2 and Dsc2 knockdown-induced malignant behaviors were elucidated using western blotting assay as well as three inhibitors including MK2206 for AKT, PD98059 for ERK, and XAV-939 for β-catenin.

**Results:**

We found reduced expressions of Dsg2 and Dsc2 in human BC tissues and cell lines compared to normal counterparts. Furthermore, shRNA-mediated downregulation of Dsg2 and Dsc2 could significantly enhance cell proliferation, migration and invasion in triple-negative MDA-MB-231 and luminal MCF-7 BC cells. Mechanistically, EGFR activity was decreased but downstream AKT and ERK pathways were both activated maybe through other activated protein tyrosine kinases in shDsg2 and shDsc2 MDA-MB-231 cells since protein tyrosine kinases are key drivers of triple-negative BC survival. Additionally, AKT inhibitor treatment displayed much stronger capacity to abolish shDsg2 and shDsc2 induced progression compared to ERK inhibition, which was due to feedback activation of AKT pathway induced by ERK inhibition. In contrast, all of EGFR, AKT and ERK activities were attenuated, whereas β-catenin was accumulated in shDsg2 and shDsc2 MCF-7 cells. These results indicate that EGFR-targeted therapy is not a good choice for BC patients with low Dsg2 or Dsc2 expression. Comparatively, AKT inhibitors may be more helpful to triple-negative BC patients with low Dsg2 or Dsc2 expression, while therapies targeting β-catenin can be considered for luminal BC patients with low Dsg2 or Dsc2 expression.

**Conclusion:**

Our finding demonstrate that single knockdown of Dsg2 or Dsc2 could promote proliferation, motility and invasion in triple-negative MDA-MB-231 and luminal MCF-7 cells. Nevertheless, the underlying mechanisms were cellular context-specific and distinct.

**Supplementary Information:**

The online version contains supplementary material available at 10.1186/s12885-024-12229-2.

## Background

Desmogleins and desmocollins belong to “non-classical” desmosomal cadherin family members that constitute the adhesive interface at the core domain of desmosome junctions. Four desmoglein (Dsg1–4) and three desmocollin isoforms (Dsc1–3) have been found to be expressed in a tissue- and differentiation-specific manner in human [1–3]. Their cytoplasmic domains bind to plakoglobin (γ-catenin), a close homolog of β-catenin, to assist in linking the desmosomes with the intermediate filament cytoskeleton [4]. Dsg2 and Dsc2 are ubiquitous in desmosome-bearing tissues including simple epithelia, while the other isoforms are primarily expressed in stratified epithelia. Dsg2 and Dsc2 are regarded as partners since they act in the way of heterophilic interactions through opposite-charge attraction [5, 6]. It was reported that deregulation of Dsg2 and its partner Dsc2 contributed to initiation and progression of cancers in a context-dependent manner. They have oncogenic function in some cancers, but in others they can function as suppressor genes. For instance, decreased Dsg2 expression indicated poor prognosis in gallbladder carcinoma, pancreatic cancer, melanoma, gastric cancer and anaplastic thyroid cancer [7–11]. In contrast, higher Dsg2 expression was associated with progression in cancers of the lungs, the liver, the colon and the skin [12–15]. Similarly, decreased expression of Dsc2 promoted tumorigenic behavior in colorectal carcinoma, pancreatic ductal carcinoma and esophageal squamous cell carcinoma [16–18]. However, little is known about roles of Dsg2 and Dsc2 on tumorigenesis and progression in BC, which is the most common cancer among women.

Activated EGFR pathway contributes to onset and progression in many cancers including BC [19, 20]. Previous studies identified Dsg2 loss induced a reduction of EGFR phosphorylation associated with activation in carcinoma cells of colon, gallbladder, skin and the lungs [7, 12, 14, 21, 22]. But interestingly, there were different effects of Dsg2 loss on downstream pathways of EGFR activation, malignancy and resistance to EGFR-targeted therapy in these cancers.

BC is a complex and highly heterogeneous disease with several intrinsic molecular subtypes including luminal, triple-negative and some other subtypes according to ER, PR and HER2 status [23, 24]. In normal mammary gland, Dsg2 and Dsc2 are expressed in both of luminal and myoepithelial cells, and they are required for luminal cell-cell adhesion and mammary epithelial morphogenesis [25, 26]. This study aimed to investigate the effects of partner desmosomal cadherins Dsg2 and Dsc2 on the malignancy of BC and their underlying mechanisms, especially relating to EGFR and its main downstream pathways. Firstly, we found expression levels of Dsg2 and Dsc2 in BC tissues and cells were both decreased compared to normal counterparts. Next, we identified that shRNA-mediated loss of Dsg2 and Dsc2 could both promote malignant behaviors in tripe-negative MDA-MB-231 and luminal MCF-7 cells. Mechanistically, despite attenuating EGFR activation, Dsg2 or Dsc2 depletion can activate AKT and ERK pathways maybe through mediation of other tyrosine kinases in MDA-MB-231 cells, but can induce β-catenin accumulation and suppress AKT and ERK pathways in MCF-7 cells, respectively. These data demonstrate that Dsg2 and Dsc2 play important roles in regulating tumor-relevant signaling in a context-dependent manner beyond their function as cell adhesion molecules in BC.

## Materials and methods

### The bioinformatics analysis

We utilized UALCAN (http://ualcan.path.uab.edu/analysis.html), TNMplot (https://tnmplot.com/analysis/) and Kaplan-Meier Plotter (http://kmplot.com/analysis/) to analyse the mRNA expressions of Dsg2 and Dsc2 as well as their associations with progression and survival in BC [27–29].

### Human tissue specimens

A total of 30 human invasive ductal BC with adjacent normal tissues were collected from Pathology Department, Tianjin Medical University. The tissue collection and analysis conducted in this study was approved by the Ethical Committee of Tianjin Medical University, China. All 30 cases were randomly selected and were made anonymous to us. The pathologic diagnosis was counterchecked by two senior pathologists according to the 2003 World Health Organization histological classification of breast tumors. Immunohistochemistry was evaluated based on the percentage of positive staining (0: 0–10%, 1: 10–25%, 2: 26–50% and 3: >50%) and staining intensity (0: none, 1: weak, 2: intermediate, and 3: strong). The percentage and intensity scores were added to obtain an overall score. The protein expression with an overall score of 0–2 was considered “negative”, while that with an overall score of 3–6 was considered “positive”.

### Cell culture and transfection

The cell lines used in this study were HEK293T, the non-tumorigenic human breast epithelial cell line MCF10A and human BC cell lines MDA-MB-231 and MCF-7. All cell lines were obtained from the ATCC and then underwent verification using the short tandem repeat method. MCF10A was cultured in DMEM/F-12 (KeyGEN Bio TECH) with 5% FBS (NEWZERUM ), 10 µg/ml insulin, 20ng/ml EGF, 0.5 µg/ml hydrocortisone, 100ng/ml cholera toxin and 1% penicillin-streptomycin. The other three cell lines were cultured in DMEM (KeyGEN Bio TECH) supplemented with 10% FBS (NEWZERUM) and 1% penicillin-streptomycin. All cells were cultured at 37 °C with 5% CO_2_. Cells were incubated in serum-free DMEM for 24 h prior to treatment with EGF (10 ng/ml; Gibco) for another 24 h.

The plasmids were synthesized by Genecopoeia, including four short hairpin RNAs (shRNAs) targeting Dsg2, three shRNAs targeting Dsc2, and 2 non-targeting Control shRNAs. HEK293T cells were used for lentivirus packaging according to the manufacturer’s instructions (Lenti-PacTM HIV Expression Kit, Genecopoeia). The virus was added to BC cells along with 8 µg/ml polybrene. After 20 h, the medium was removed and replaced with fresh medium containing 2 µg/ml and 0.8 µg/ml of puromycin in MDA-MB-231 and MCF-7 cells, respectively. Puromycin-resistant clones which were selected by culturing for another 2 weeks in the presence of puromycin were regarded as stably transfected cells.

### Western blotting

Cells were lysed in RIPA buffer supplemented with proteinase and phosphatase inhibitors. Protein concentration was determined by ultraviolet spectrophotometer. Then equal amounts of proteins were separated by SDS-PAGE and then electrotransferred to PVDF membrane. After blocking in TBST with 5% non-fat milk, the membranes were cut into strips and then incubated overnight with various primary antibodies at 4 °C, followed by incubation with a secondary antibody (1:3000 dilution) at room temperature for 2 h. The primary antibodies were against Dsg2 (1:1000, #21880, Proteintech), Dsc2 (1:1000, #13876, Proteintech), cyclin D1 (1:5000, #60186, Proteintech), N-cadherin (1:1000, #PTM-5221, PTMBIO), E-cadherin (1:500, #7870, Santa Cruz Biotechnology), CD133 (1:500, #ab226355, Abcam), β-catenin (1:5000, #ab32572, Abcam), EGFR (1:500, #373746, Santa Cruz Biotechnology), p-EGFR (Y845) (1:500, #ab97613, Abcam), p-EGFR (Y1092) (1:1000, #PTM-6870, PTMBIO), p-ERK1/2 (T202/Y204) (1:2000, #4370, Cell Signaling Technology), p-AKT (S473) (1:1000, #PTM-6649, PTMBIO), and GAPDH for internal reference (1:3000, #TA-08, ZS Bio).

### Plate colony-forming assay

5 × 10^2^ cells were seeded into 6-well plates. Cells were maintained at 37 °C in an incubator containing 5% CO_2_. After incubation, the medium was renewed and the plates were incubated for 14 days under the same culture conditions. The inhibitors were added on the 7th day after cell seeding. The cells were washed twice with ice-cold phosphate-buffered saline (PBS) and fixed with methanol for 20 min in room temperature, followed by staining with 0.5% crystal violet.

### MTT assay

Human BC cells (1 × 10^4^/well) were placed in 96-well plates and continually cultured for different periods of time (1, 2, 3, 4 or 5 days). Subsequently, 50 µl of 0.5 mg/ml MTT (Catalog no. KGA9301, KeyGEN Bio TECH) was added to each well. The cells were incubated at 37 °C for another 4 h, the medium was removed, and the precipitated formazan was dissolved in 100 µl of DMSO. After the solution was shaken for 10 min using an Eppendorf Mix Mate (Eppendorf, GRE), the absorbance was detected at 490 nm (A490) on a Bio Tek ELx800 (Bio Tek, USA).

### Wound-healing assay

Cells of each groups were incubated in 6-well plates. The cell monolayer was scraped in a straight line with 100 µl pipette tip and incubated with serum free medium. Photographs of scratches were monitored by an invert microscope at different time points. Cell motility was assessed by measuring the migration of cells into a scrape. The speed of wound closure was monitored after 48 and 72 h by measuring the ratio of the area size of the wound relative to that at hour 0. Each experiment was performed in triplicate.

### Transwell migration and invasion assays

Transwell migration and invasion assays were performed with an 8.0 μm pore filter chamber (Invitrogen) without or with Matrigel inserted in 24-well plates. The BC cells (1 × 10^5^ cells) in 100 µl of DMEM without FBS were seeded into the upper wells, and DMEM with 10% FBS were added to the bottom chamber. Transfected MDA-MB-231 cells were incubated and allowed to migrate for 24 h or invade through the Matrigel for 48 h. Transfected MCF-7 cells were incubated for an additional 24 h. After fixing with methanol, the non-invading cells were removed from the upper surface. The invaded cells adhering to the bottom surface of the membrane were stained with 0.5% crystal violet. Using an inverted light microscope (Nikon), we counted the number of invading cells. All experiments were repeated independently at least three times.

### Immunofluorescence staining

The cells were tiled on coverslips, incubated at 37 °C overnight, fixed with ethanol and blocked with 5% FBS. Then, the cells were incubated with primary antibodies against Dsg2 (#21880; Proteintech; 1:50) and Dsc2 (#13876; Proteintech; 1:50). After incubation with fluorophore-conjugated secondary antibodies, the nuclei were counterstained with 4’,6-diamidino-2-phenylindole(DAPI) (Sigma). Images were acquired by fluorescence microscopy (Nikon, Japan).

### Statistical analysis

For all analysis, mean ± SD of the measurements was calculated and illustrated in the histograms or broken line graphs. Student’s t-test and chi-square analysis were used to determine whether there was a significant difference between two means, and nonparametric ANOVA was performed for comparison of multiple means. The data were analysed with the GraphPad Prism 8.0 (GraphPad Software). P values (two-sided) less than 0.05 were considered statistically significant.

## Results

### Both Dsg2 and Dsc2 were down regulated in BC tissues and cells

**Fig. 1:**
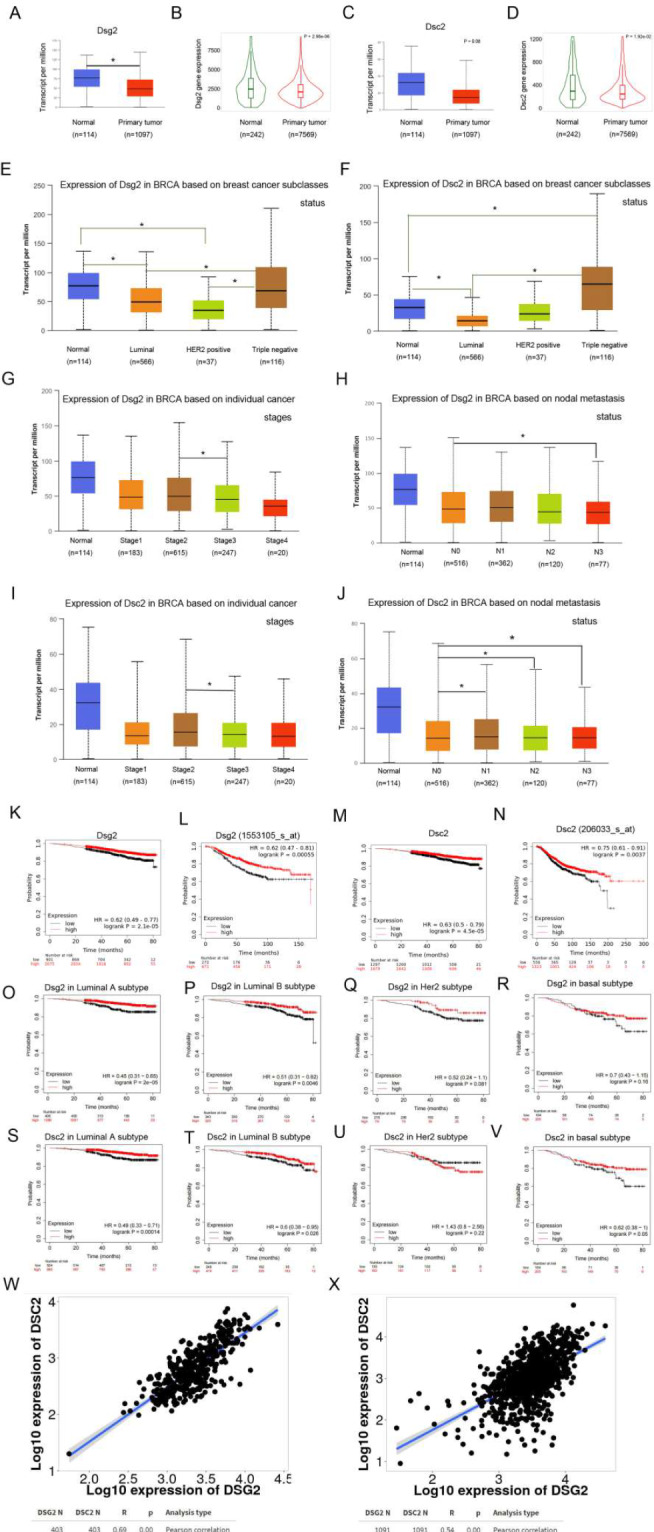
The low mRNA expressions of Dsg2 and Dsc2 were associated with progressionand poor prognosis by bioinformatics analysis. (**A**-**D**) The mRNA expressions of Dsg2 and Dsc2 were reduced in BC tissues from TCGA database (**A**, **C**) and TNMplot database (**B**, **D**). (**E**-**F**) The mRNA expressions of Dsg2 and Dsc2 were associated with molecular subtypes of BC from TCGA database. (**G**-**J**) The mRNA expression levels of Dsg2 and Dsc2 were negatively correlated with stage and nodal metastasis status of BC from TCGA database. (**K**-**V**) KM plotter analysis showed the low mRNA expressions of Dsg2 and Dsc2 were associated with poor overall survival whatever in all population or in molecular subtypes. (**W**-**X**) The mRNA expressions of Dsg2 and Dsc2 were positively correlated in normal breast tissues (**W**) and in BC tissues from the TNMplot data. *indicates *P* < 0.05

Firstly, we utilized web tools to investigate the associations of Dsg2 and Dsc2 with BC. We found that BC tissues expressed lower Dsg2 and Dsc2 mRNA levels compared with normal tissues in both TCGA and TNMplot databases (Fig. 1A-D). Both Dsg2 and Dsc2 at mRNA level were higher in TNBC molecular subtype than that in the HER2 and luminal subtypes (Fig. 1E-F). In addition, Dsg2 and Dsc2 mRNA levels were both negatively associated with individual cancer stages and nodal metastasis status of BC in TCGA database. The levels of Dsg2 and Dsc2 were both reduced significantly in stage 3 than that in stage 2 according to AJCC pTNM stage. In N3 status that meant metastases in 10 or more axillary lymph nodes there was markedly lower Dsg2 level than that in N0 that meant no regional lymph node metastasis. For Dsc2, there were significant reductions in each of N1, N2 and N3 status compared to that in N0 status, respectively (Fig. 1G-J). Moreover, Kaplan-Meier Plotter analysis showed that low Dsg2 and Dsc2 mRNA levels were associated with poor overall survival in both gene chip and RNA-seq data (Fig. 1K-N). The similar overall survival results of Dsg2 and Dsc2 were exhibited in four molecular subtypes, except that Dsc2 played in HER2 subtype (Fig. 1O-V). The last part of bioinformatics analysis was to examine the correlation between Dsg2 and Dsc2 with the help of TNMplot data. In the normal breast tissues, the correlation coefficient was 0.69 while it became 0.54 in the invasive breast cancers, which suggested the correlation was a little weaker in BC compared to that in normal tissues (Fig. 1W-X).

**Fig. 2:**
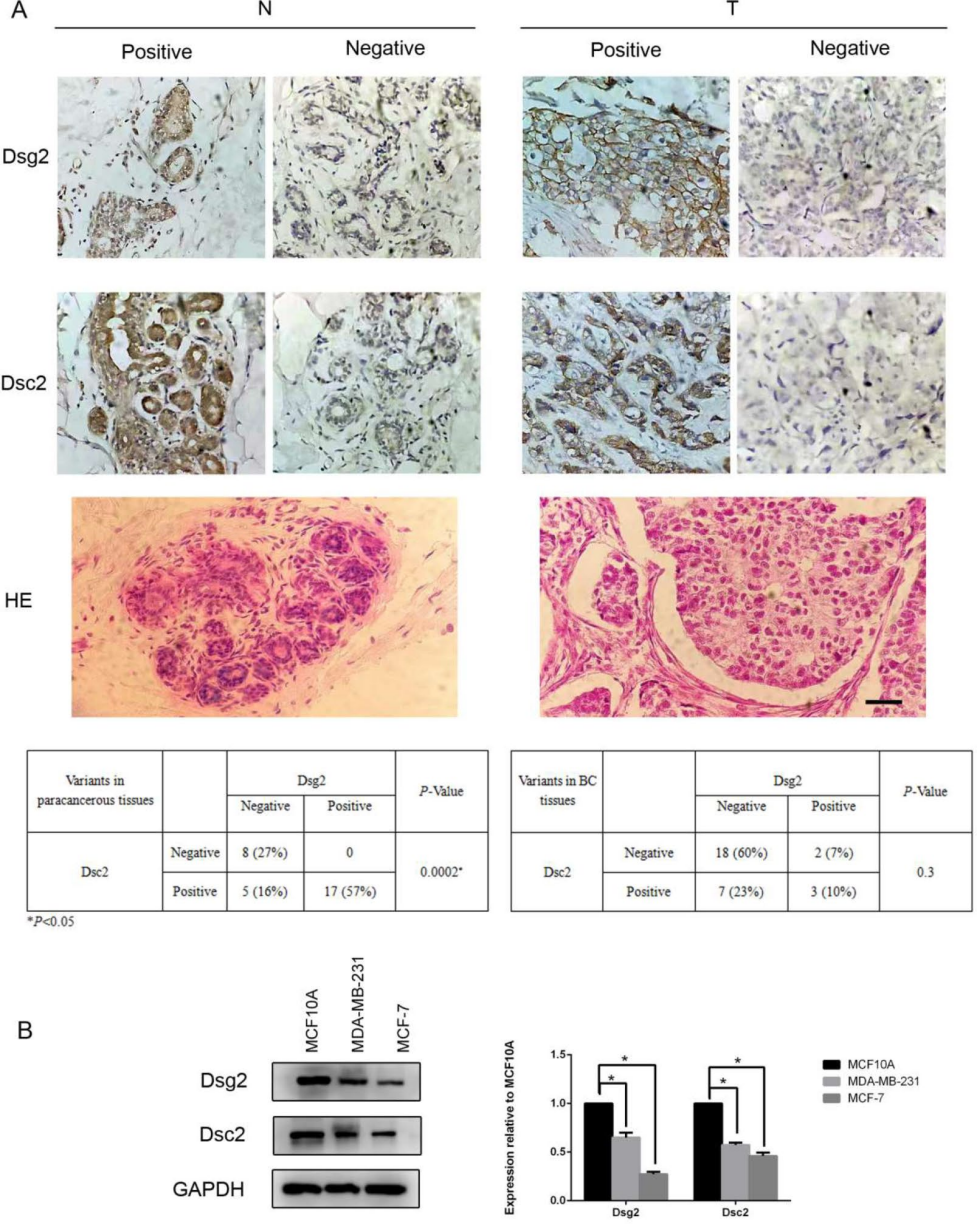
The protein levels of Dsg2 and Dsc2 were decreased in BC tissues and cell lines. (**A**) Protein expressions of Dsg2 and Dsc2 in 30 paired BC and adjacent normal tissues analyzed by IHC; T (Tumor), N (Normal). Scale bar represents 50 μm. (**B**) Protein expressions of Dsg2 and Dsc2 in BC cell lines and non-tumorigenic human breast MCF10A cells, GAPDH as the loading control. Data are presented as mean ± SD from three separate experiments. *indicates *P* < 0.05

We then collected 30 pairs of BC tissues and the paracancerous normal tissues and examined the protein expressions of Dsg2 and Dsc2 by IHC. Dsg2 and Dsc2 were both located at the cell membrane and in the cytoplasm. In paracancerous normal tissues, positive Dsg2 and Dsc2 expressions were observed in 57% and 73% of cases, respectively. Indeed, there was a positive correlation between Dsg2 and Dsc2 expression. In contrast, the percentage of Dsg2 and Dsc2 positive expression dropped to 17% and 33% in BC, respectively. Meanwhile, the correlation between Dsg2 and Dsc2 expression became not significant statistically in BC (Fig. 2A).

Then we examined protein levels of Dsg2 and Dsc2 of human BC cells MDA-MB-231 and MCF-7 cells and human non-neoplastic mammary epithelial cells, MCF10A by western blot. The results revealed that both Dsg2 and Dsc2 were dramatically down regulated in both BC cell lines compared to MCF10A, which was similar to that in human tissues (Fig. 2B).

### Dsg2 and Dsc2 downregulations both significantly facilitated proliferation, migration and invasion in BC cells

**Fig. 3:**
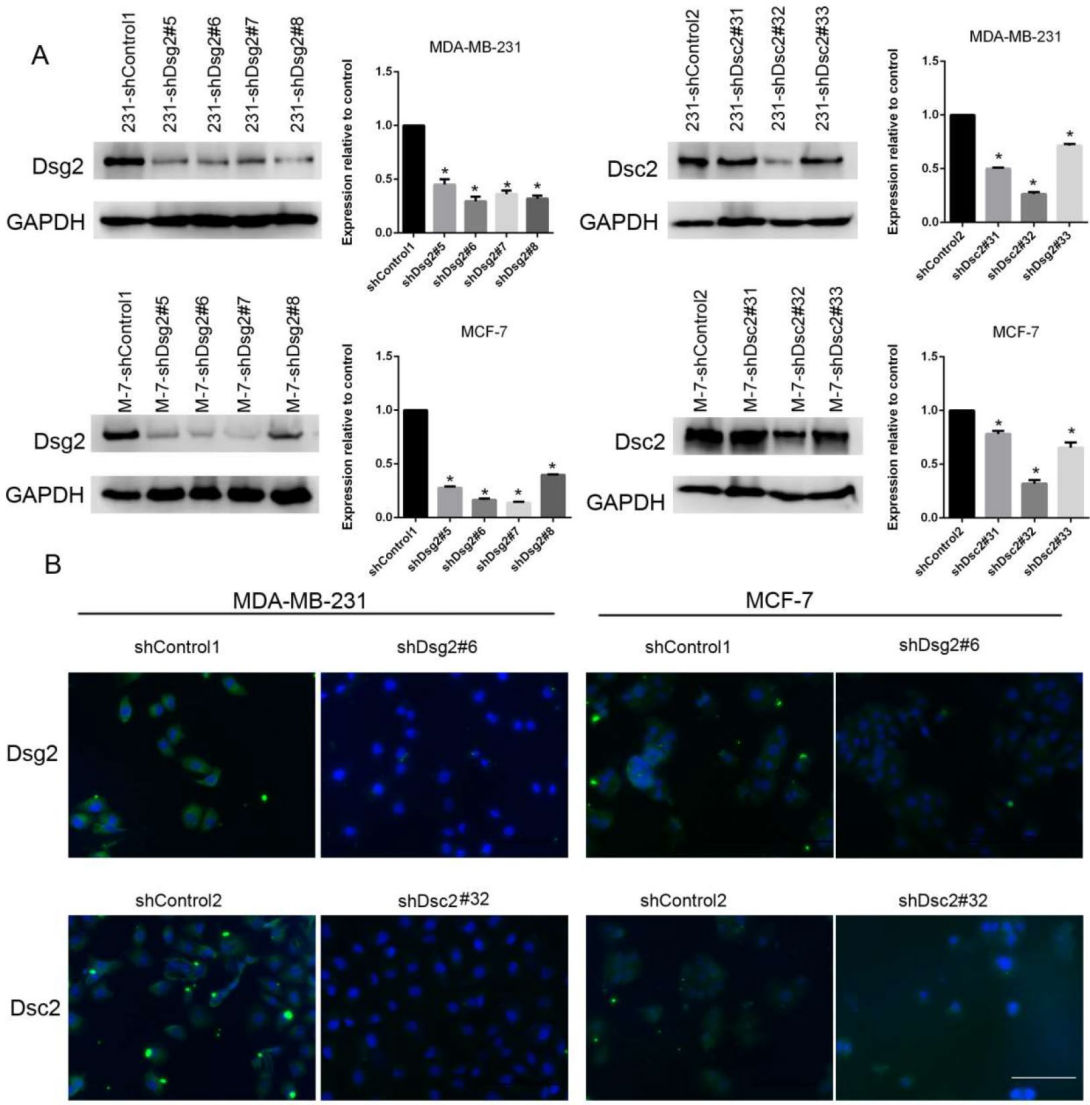
Identification of shRNA-mediated stable knockdown of Dsg2 and Dsc2 in BC cell lines. (**A**) The comparison of protein levels from four clones (#5, #6, #7 and#8) of shDsg2 and three clones (#31, #32 and#33) of shDsc2 with their controls (shControl1 and shControl2) in MDA-MB-231 cells and MCF-7 cells by western blot, respectively. (**B**) The further confirmation of knockdown efficiency of clones #6 and #32 by immunofluorescence. Scale bar represents 100 μm. All experiments were repeated three times; Data are presented as mean ± SD from the three separate experiments. *indicates *P* < 0.05

To further evaluate the biological roles of reduced Dsg2 and Dsc2 in BC with different subtypes, we generated stable knockdown MDA-MB-231 and MCF-7 cell lines expressing Dsg2- specific (shDsg2), Dsc2- specific (shDsc2) and non-targeting short hairpin RNA (shRNA) controls (shControl1 and shControl2) in parallel via lentivirus transduction, respectively. Four clones of shDsg2 and three clones of shDsc2 for both of the cell lines were produced, respectively. As shown in Fig. 3A and B, clones shDsg2#6 and shDsc2#32 which exhibited the best downregulation effect by western blot and then were confirmed by immunofluorescence were selected for the subsequent experiments.

**Fig. 4:**
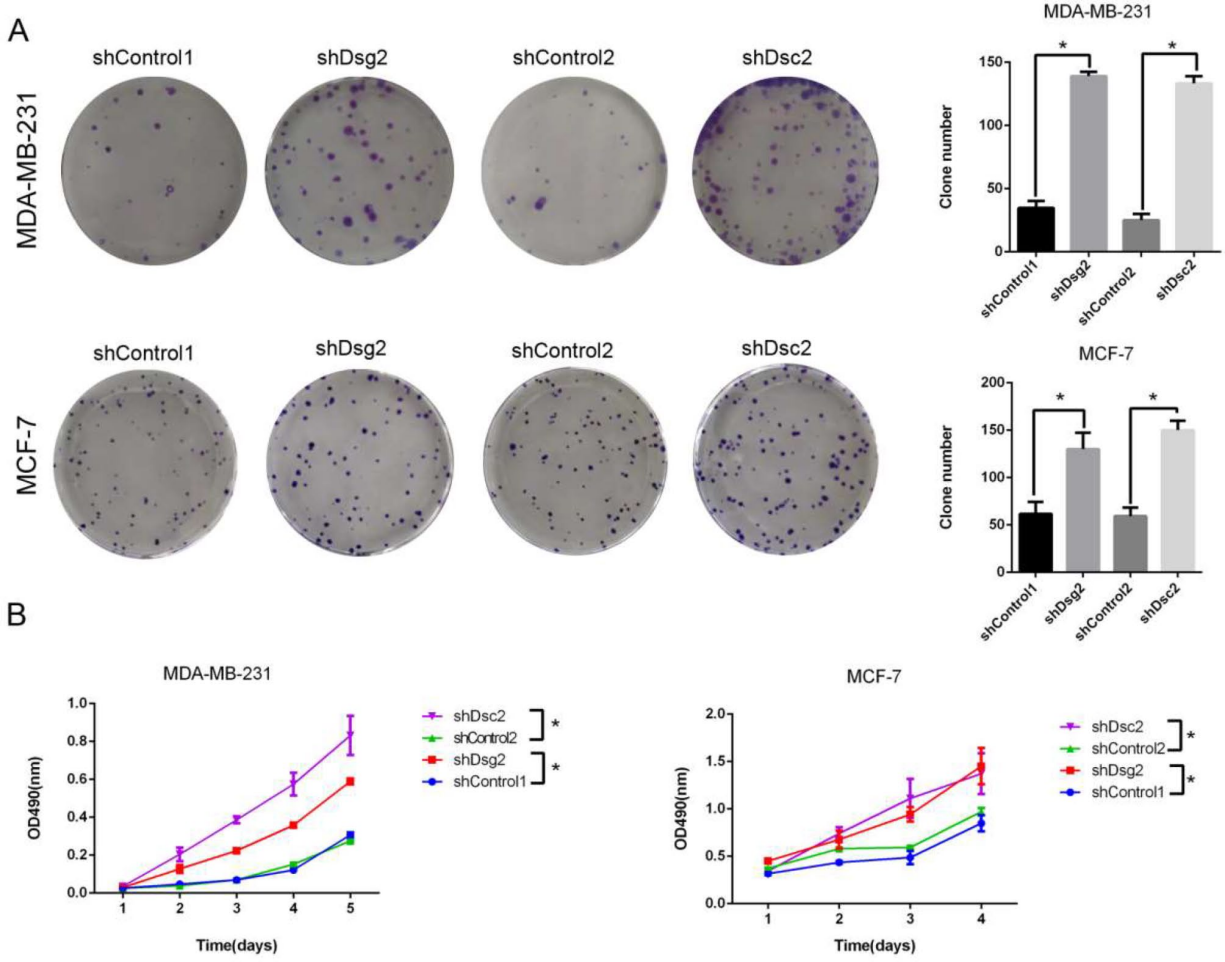
Dsg2 and Dsc2 loss both promoted proliferation of BC cells. (**A**) Plate colony-forming assays were performed in the indicated groups, and the number of colonies was calculated by Image J. (**B**) The viability and proliferation were examined by MTT assays. Data are presented as mean ± SD from three separate experiments. *indicates *P* < 0.05

Firstly, we performed single-cell plate colony-forming assay to examine the effect of Dsg2 and Dsc2 loss on proliferation capacity of BC cells. We observed that the number and size of colonies formed were increased in Dsg2- or Dsc2-depleted BC cells compared with that in the control groups (Fig. 4A). Second, in the performed MTT assay, Dsg2- or Dsc2- silencing BC cells displayed higher OD values than controls (Fig. 4B). These results consistently indicated that Dsg2 and Dsc2 knockdown significantly facilitated the proliferation of both MDA-MB-231 and MCF-7 cell lines.

**Fig. 5:**
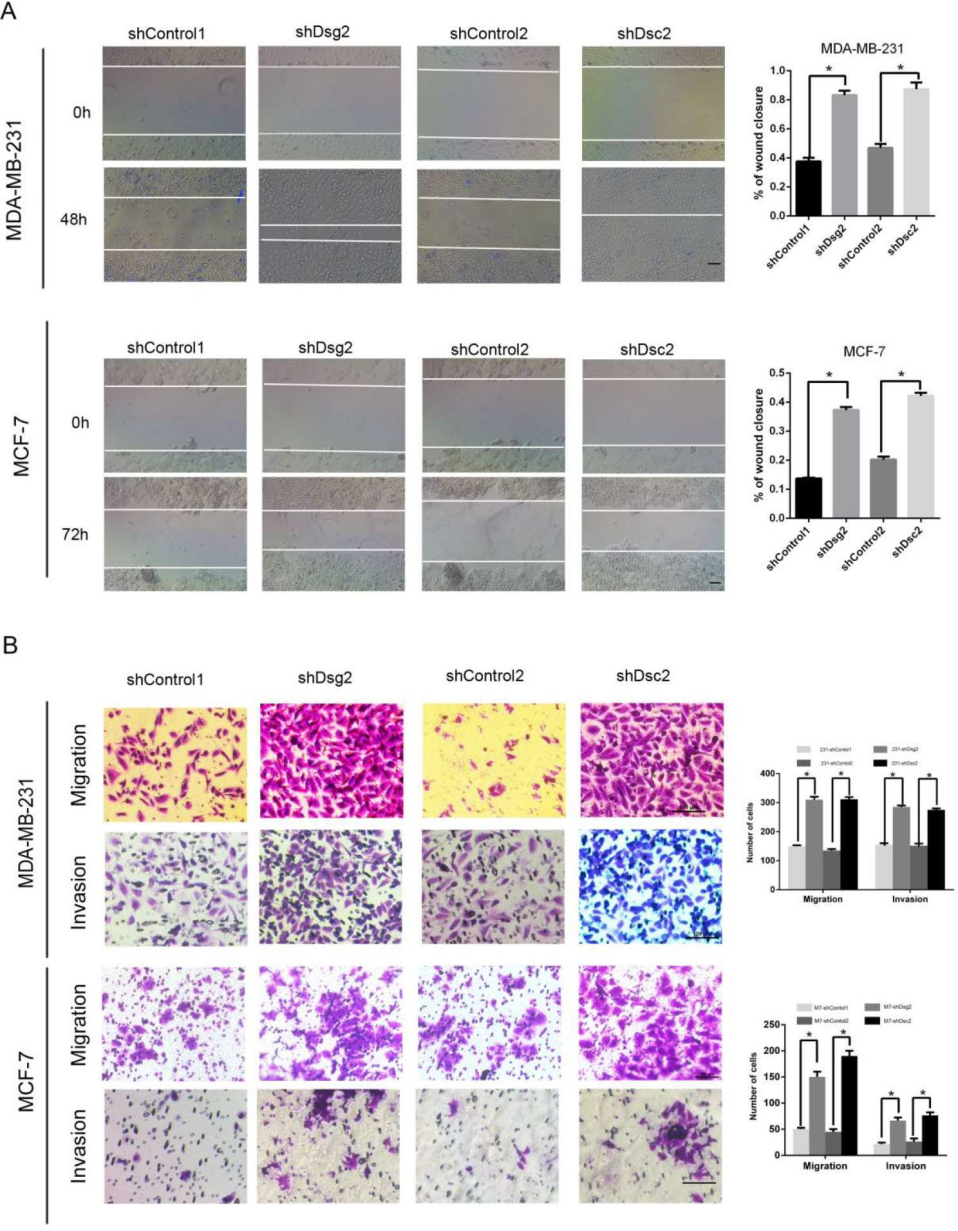
Dsg2 and Dsc2 depletions both enhanced migration and invasion of BC cells. (**A**) Wound-healing assays and quantitative analysis showed a significant promotion of migration at 48 h for shDsg2 and shDsc2 MDA-MB-231 cells and at 72 h for shDsg2 and shDsc2 MCF-7 cells compared with their controls. (**B**) Transwell migration and invasion assays. The images of migrated cells were photographed after 24 h for seeding derived cells from MDA-MB-231 cells and 48 h for seeding derived cells from MCF-7 cells. The images of invaded cells were photographed at 48 h for MDA-MB-231 cells and 72 h for MCF-7 cells. The scale bar represents 100 μm. Data are presented as mean ± SD from three separate experiments. *indicates *P* < 0.05

In addition, wound-healing assay and transwell migration assay were performed to determine whether Dsg2 and Dsc2 silencing may regulate cell motility in BC cells. In wound-healing assay we observed a significant increase in the rate of wound closure of both cell monolayers with reduced Dsg2 or Dsc2 levels (Fig. 5A). Similarly, transwell migration assay showed downregulation of Dsg2 or Dsc2 significantly increased the amount of cells that passed the chamber without matrigel (Fig. 5B). Besides, consistent results were observed in the matrigel invasion assay (Fig. 5B). These results suggested Dsg2 or Dsc2 loss could promote migration and invasion in both MDA-MB-231 and MCF-7 cells.

**Fig. 6:**
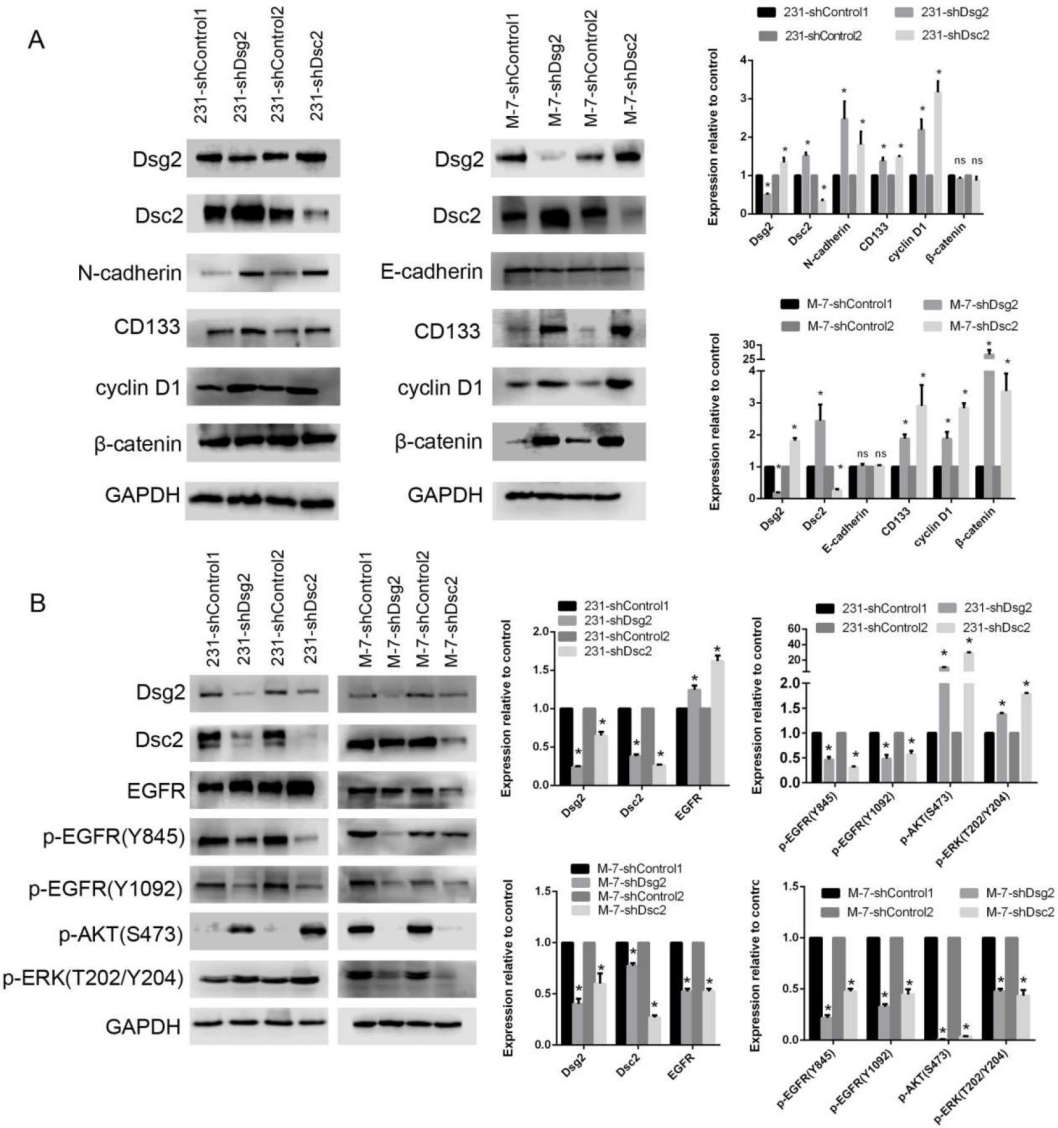
The expressions of function-associated proteins and pathway-associated proteins were determined in shDsg2 and shDsc2 BC cells by western blot. (**A**) Cells were lysed and lysates subjected to western blot to detect N-cadherin, E-cadherin, CD133, cyclin D1 and β-catenin with the respective antibodies. (**B**) Cells were stimulated with 10 ng/ml EGF for 24 h after starved in serum-free DMEM for 24 h. Subsequently, the cells were lysed and western blot was performed using antibodies against EGFR pathways. Data are presented as mean ± SD from three separate experiments. *indicates *P* < 0.05; ns, not significant

Moreover, malignancy associated proteins N-cadherin, CD133 and cyclin D1 were dramatically increased but β-catenin was almost unchanged in Dsg2- or Dsc2- depleted MDA-MB-231 cells by western blot. However, Dsg2- or Dsc2- depleted MCF-7 cells displayed higher levels of CD133, cyclin D1 and β-catenin, but E-cadherin had no obvious alteration (Fig. 6A). These data indicated there must be some distinct underlying mechanisms by which Dsg2 or Dsc2 loss enhanced malignancy in the tripe-negative MDA-MB-231 cells and luminal MCF-7 cells.

### Silencing of Dsg2 and Dsc2 could both activate AKT and ERK signaling pathways in MDA-MB-231 cells, but inversely suppress these pathways in MCF-7 cells

We next sought to elucidate the molecular mechanism by which Dsg2 and Dsc2 loss induced progression of BC cells. Considering Dsg2 and Dsc2 affected EGFR signaling pathway in some cancers such as cancers of gallbladder, colon, cervix and the lungs, we focused on EGFR phosphorylation and its major downstream AKT and ERK pathways. As expected, phosphorylation levels of EGFR on Y845 and Y1092 that have been linked to activation were decreased in shDsg2 or shDsc2 cells compared to controls in both MDA-MB-231 and MCF-7 cell lines. But to our surprise, Dsg2 or Dsc2 depletion substantially raised the phosphorylation of AKT on S473 and ERK on T202/Y204 in MDA-MB-231 cells, but made them lowered in MCF-7 cells (Fig. 6B). These findings suggested that there must be alternative pathways which could promote proliferation and migration when EGFR activation had been attenuated in shDsg2 or shDsc2 cells, yet the alternative mechanisms were distinct in MDA-MB-231 and MCF-7 cells which had different genetic background and exhibited highly different phenotypes.

### Inhibition of AKT activity was more effective to suppress tumor progression than blockade of ERK activity in shDsg2 and shDsc2 MDA-MB-231 cells

**Fig. 7:**
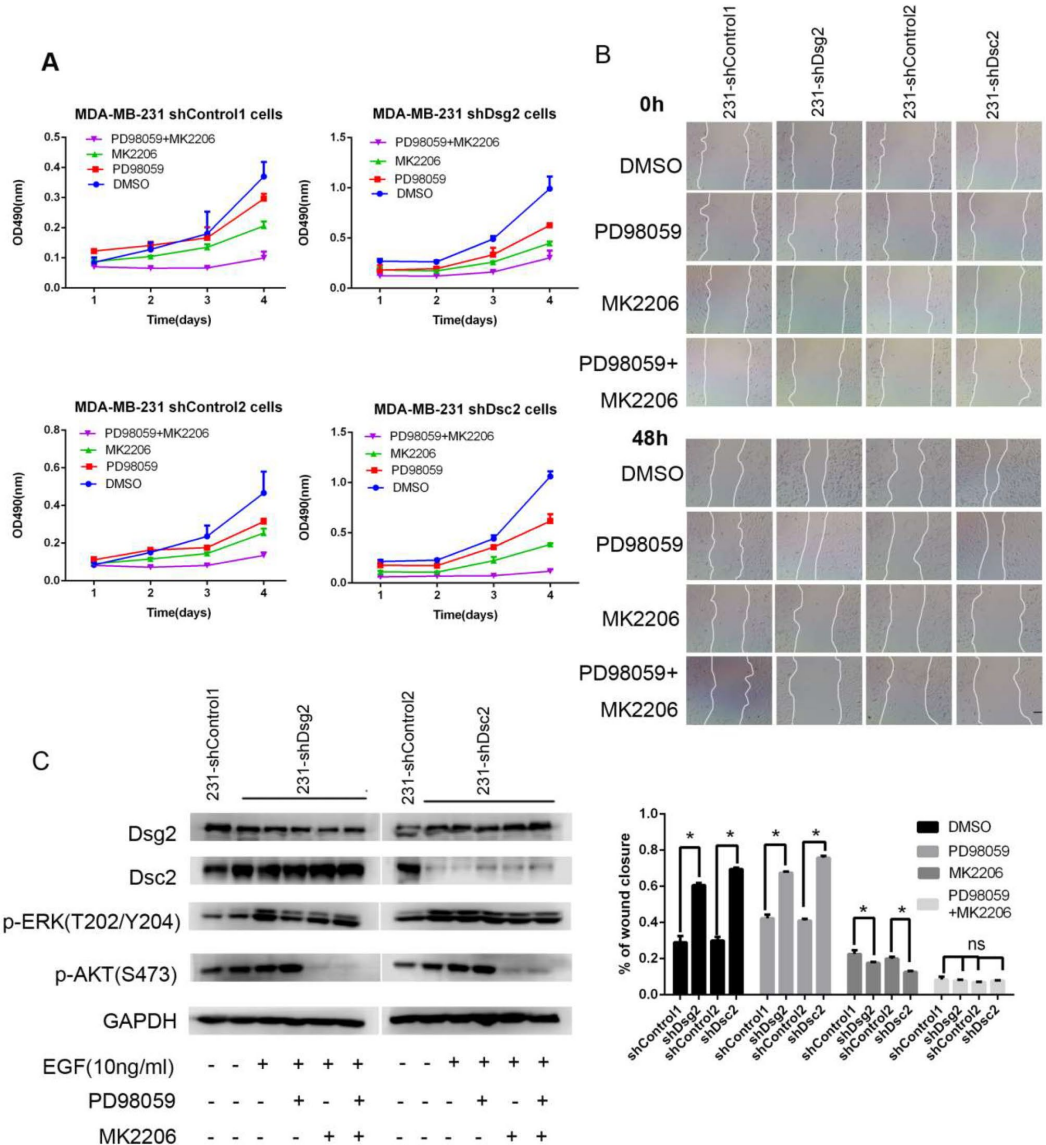
AKT inhibition demonstrated stronger anti-proliferative and anti-migratory effect than ERK inhibition in shDsg2 and shDsc2 MDA-MB-231 cells. (**A**) Inhibition of AKT activity by the inhibitor MK2206 (2µM) reduced the proliferation more notably compared to the ERK inhibitor PD98059 (20µM) by MTT assays in MDA-MB-231 cells, particularly in shDsg2 and shDsc2 MDA-MB-231 cells. (**B**) Wound-healing assays indicated that AKT inhibition reversed Dsg2 or Dsc2 depletion-induced migration, while ERK inhibition had no this effect. The scale bar represents 100 μm. (**C**) Western blot analysis of p-AKT and p-ERK expression in response to MK2206 and PD98059 treatment in shDsg2 and shDsc2 MDA-MB-231 cells. Cells were pretreated with 2 µM MK2206, 20 µM PD98059, or combination of them for 4 h and then stimulated with 10 ng/ml EGF for 24 h. Cell lysates were collected and analyzed by western blot using the indicated antibodies. Data are presented as mean ± SD from three separate experiments. *indicates *P* < 0.05; ns, not significant

Since both p-AKT and p-ERK were increased in shDsg2 and shDsc2 MDA-MB-231 cells, we wondered whether inhibition of AKT and ERK pathways could reverse malignant behaviors enhanced by Dsg2 or Dsc2 loss. We treated shDsg2 and shDsc2 MDA-MB-231 cells and their controls with inhibitor of AKT (MK2206, 2 µM), inhibitor of ERK (PD98059, 20 µM) or both of them at a concentration of inhibitor which did not significantly affect cell growth. MTT assays and single-cell plate colony-forming assays revealed MK2206 displayed much stronger ability to inhibit proliferation compared with PD98059 among MDA-MB-231 cells with different Dsg2 or Dsc2 expression (MTT data in Fig. 7A; plate colony-forming data not shown). In addition, wound-healing assays suggested MK2206 dramatically suppressed motility particularly in shDsg2 and shDsc2 MDA-MB-231 cells. In contrast, ERK inhibitor PD98059 slightly abated proliferation but did not repress the enhanced migration in shDsg2 and shDsc2 MDA-MB-231 cells, which could be interpreted by a significant increase of p-AKT owing to ERK inhibition (Fig. 7B and C). Altogether, these data indicated that AKT inhibition was more powerful to suppress Dsg2- and Dsc2-depletion induced progression than ERK inhibition in MDA-MB-231 cells.

### Accumulation of β-catenin was an alternative mechanism for decreased AKT and ERK activities in shDsg2 or shDsc2 MCF-7 cells

**Fig. 8:**
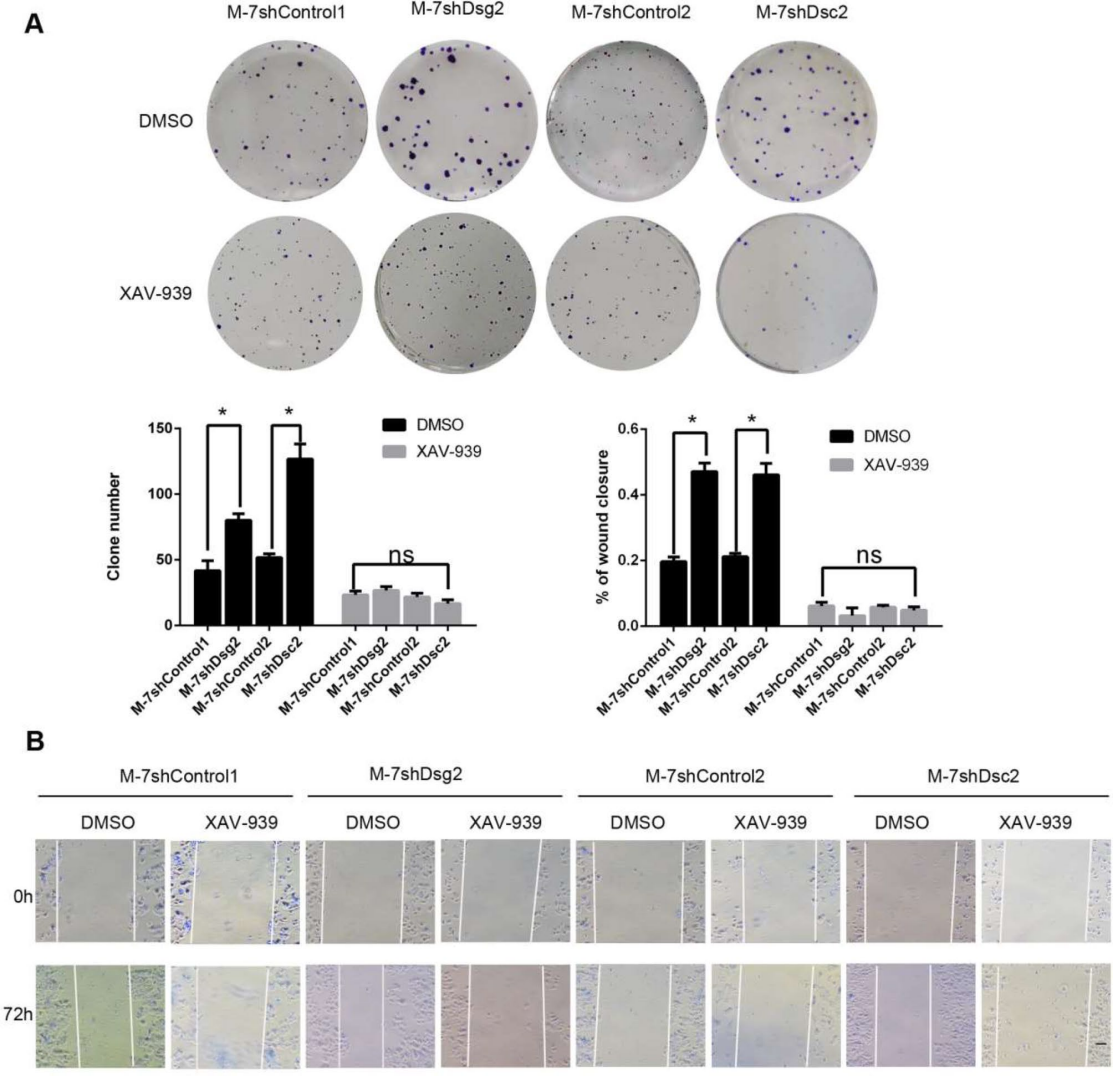
XAV-939 abolished shDsg2- and shDsc2- induced proliferation and migration in MCF-7 cells. (**A**) Plate clone-forming assays showed β-catenin inhibitor XAV-939 (5µM) could attenuate capacity of proliferation in shDsg2 and shDsc2 MCF-7 cells. (**B**) Wound-healing assay displayed XAV-939 (5µM) abrogated shDsg2- and shDsc2- induced migration in MCF-7 cells. The scale bar represents 100 μm. Data are presented as mean ± SD from three separate experiments. *indicates *P* < 0.05; ns, not significant

Although the AKT and ERK activities were both suppressed in shDsg2 or shDsc2 MCF-7 cells, cell’s capacity of proliferation, motility and invasion was enhanced. Therefore, we speculated that there were some other signaling pathways activated to compensate for decreased AKT and ERK activities. Considering loss of Dsc2 activated β-catenin signaling and promoted progression in an E-cadherin-dependent manner in oesophageal squamous cell carcinoma, we focused on expression of β-catenin and E-cadherin in shDsg2 and shDsc2 MCF-7 cells. As shown in Fig. 6A, protein level of E-cadherin did not obviously alter but β-catenin was increased markedly in shDsg2 or shDsc2 MCF-7 cells. When we added inhibitor of β-catenin (XAV-939, 5 µM), the enhanced capacity of proliferation and motility was abolished. (Fig. 8). These data indicated that loss of Dsg2 or Dsc2 could promote proliferation, motility and invasion through β-catenin accumulation in MCF-7 cells expressing high E-cadherin.

### The expression dependence between Dsg2 and Dsc2 in BC

Since Dsg2 and Dsc2 downregulation could both facilitate malignant progression and share the same regulatory mechanism, we wondered the impact on Dsc2 expression of Dsg2 knockdown and vice versa in BC cells. Hence, we examined Dsc2 protein level in shDsg2 cells and Dsg2 protein level in shDsc2 cells by western blot, respectively. The first several results after transfection showed the higher expression of the other partner desmosomal protein when one was knockdown (Figs. 6A and 7C). However, it was decreased when the western blot was performed after stably transfected cells were cultured for approximately more than one month (Fig. 6B). Therefore, we speculated that the increased expression was transient compensatory response to the decreased partner at early stage, and the decreased expression was adaptive response to the sustained lower partner.

Taken together, single downregulation of the partner desmosomal cadherins Dsg2 or Dsc2 can promote proliferation, migration and invasion, and they share the same signaling pathways.

## Discussion

Cancer initiation and progression are complex progresses in which many signaling pathways are involved. Crosstalk between different pathways allows the integration of the great diversity of stimuli that a cell receives [30–33]. As cytomembrane proteins, desmosomal cadherins and EGFR, a common receptor tyrosine kinase (RTK), have been reported to interact in several cancers previously [21, 22, 34]. EGFR pathway plays important roles in cancers including BC [20, 35]. In this study, we simultaneously investigated the roles of two common partner desmosomal cadherins Dsg2 and Dsc2 played in BC as well as their effect on EGFR pathway. We found shRNA mediated-knockdown of Dsg2 and Dsc2 could both enhance proliferation, migration and invasion in triple-negative MDA-MB-231 and luminal MCF-7 cells. However, the phosphorylation levels of EGFR on Y845 and Y1092, two representatives of EGFR activation, were reduced upon loss of Dsg2 or Dsc2 whatever in triple-negative MDA-MB-231 cells expressing higher EGFR or in luminal MCF-7 cells with lower EGFR level [36]. EGFR phosphorylation on Y845 is usually catalyzed by Src and Y1092 belongs to auto-phosphorylation sites. Y845 and Y1092 phosphorylation-mediated signaling is linked to higher cancer malignancy due to enhanced cell transformation, proliferation, motility, and invasion [37, 38]. It is obvious that decreased phosphorylation of EGFR on Y845 and Y1092 could not account for increased malignancy in our study. We speculate that there must be other alternative signaling pathways enhancing malignant behaviors. However, our data revealed the alternative pathways were obviously distinct in MDA-MB-231 and MCF-7 cells partly due to their different cellular genetic background [39].

In triple-negative MDA-MB-231 cells, the p-AKT and p-ERK, two of the main downstream molecules of EGFR pathway were increased upon loss of Dsg2 or Dsc2. These results are similar to that in both gallbladder carcinoma and anaplastic thyroid cancer reported by Jeong-Ki Min previously [7, 11]. Jeong-Ki Min et al. found loss of Dsg2 promoted gallbladder carcinoma progression and resistance to EGFR-targeted therapy through Src kinase activation, a non-receptor tyrosine kinase (nRTK), by which increased p-AKT and p-ERK were involved, indicating PI3K and MAPK pathways were activated. In addition, in anaplastic thyroid cancer Dsg2 depletion significantly increased metastatic potential through activation of c-Met which belonged to receptor tyrosine kinase (RTK) and also transmitted higher downstream p-AKT activity. Besides, it is worth noting that competition exists among different RTKs for the same downstream signaling molecules [40–43]. Based on these above, we proposed that loss of Dsg2 or Dsc2 that inhibited EGFR activity may cause competitive activation of some other tyrosine kinases including nRTKs and RTKs, and then transmit positive signals to downstream AKT and ERK pathways in MDA-MB-231 cells which overexpressed and relied overly on tyrosine kinases [44]. It needs to explore which tyrosine kinases are activated by loss of Dsg2 or Dsc2 and how they are activated next. Futhermore, in our study ERK inhibition exhibited much weaker anti-tumor effect than AKT inhibition in general, which could be attributed to feedback activation of p-AKT induced by ERK inhibitor PD98059 in shDsg2 and shDsc2 MDA-MB-231 cells. Feedback activation is commonly observed in cancers and it contributes to resistance to the targeted therapy of cancer [45–47]. Therefore, inhibitors of AKT, not of ERK, can be considered for triple-negative BC patients with low Dsg2 or Dsc2 expression.

In luminal MCF-7 cells which expressed low tyrosine kinases but high E-cadherin, Dsg2 or Dsc2 depletion attenuated EGFR activity and the downstream PI3K and MAPK pathways [44]. Consistent with the result, Bing-Xia Zhou et al. showed that in cervical cancer cells transfected with si-Dsg2, p-ERK was significantly decreased [48]. Likewise, another study reported that shDsg2 colon cancer cells had decreased p-ERK and p-Src levels [14]. However, shDsg2 or shDsc2 MCF-7 cells exhibited enhanced malignancy in our study while Dsg2-deficient cervical cancer and colon cancer cells showed inhibited progression. Moreover, we found that increased β-catenin contributed to enhanced tumor malignant behaviors in MCF-7 shDsg2 or shDsc2 cells, which was consistent with that in E-cadherin-expressing oesophageal squamous cell carcinoma (ESCC) cells reported by Wang-Kai Fang et al. They found Dsc2 loss can release more free γ-catenin which may compete with β-catenin, thus displacing the latter from E-cadherin and increasing β-catenin- dependent transcriptional activity [49]. Besides, Kolegraff et al. reported that loss of Dsc2 contributed to the growth of colorectal cancer cells through the activation of AKT/β-catenin signaling [16]. MCF-7 cell, a less aggressive BC cell line, overexpresses E-cadherin, a classic cadherin. Γ-catenin and β-catenin are highly homologous. Γ-catenin can interact with E-cadherin as β-catenin but, in addition, binds to the desmosomal cadherins desmocollin and desmogelin in desmosomes. In our study, loss of Dsg2 and Dsc2 in MCF-7 cells could release more free γ-catenin to compete with its close relative, β-catenin to bind to E-caherin. In addition, loss of Dsg2 and Dsc2 might activate Wnt signaling to reduce degradation of free β-catenin. Therefore, more free β-catenin was accumulated in the cytoplasm and the nucleus. Increasing accumulation in the nucleus promoted β-catenin-dependent transcriptional activity. All these above involving MCF-7 cells are our speculation, but how Dsg2 or Dsg2 loss induced β-catenin accumulation in MCF-7 cells specifically need much in-depth study in future.

In conclusion, this study determined that both of Dsg2 and Dsc2 were down regulated in human BC tissues and cells. In vitro experiments confirmed that in spite of reducing EGFR activity, single loss of Dsg2 or Dsc2 could activate AKT and ERK pathways in MDA-MB-231 cells and facilitate β-catenin accumulation in MCF-7 cells to promote malignancy, respectively. This study suggests that AKT inhibitors can be chosen for triple-negative BC patients with low Dsg2 or Dsc2 expression while luminal BC patients with low Dsg2 or Dsc2 may benefit from inhibitors targeting β-catenin. More specific mechanisms are needed to be clarified in future.

### Electronic supplementary material

Below is the link to the electronic supplementary material.

Supplementary Material 1

## Data Availability

The data from bioinformatics analysis were originated from UALCAN (http://ualcan.path.uab.edu/analysis.html), TNMplot (https://tnmplot.com/analysis/) and Kaplan-Meier Plotter (http://kmplot.com/analysis/) open-access databases.
