# Supplementary material for: Desmoglein 2 and desmocollin 2 depletions promote malignancy through distinct mechanisms in triple-negative and luminal breast cancer
Source: BMC Cancer. 2024 Apr 26;24:532. doi: 10.1186/s12885-024-12229-2 (PMC11046749; doi:10.1186/s12885-024-12229-2)

Fig. 2B Protein expressions of Dsg2 and Dsc2 in BC cell lines and non-tumorigenic human breast MCF10A cells, GAPDH as the loading control.

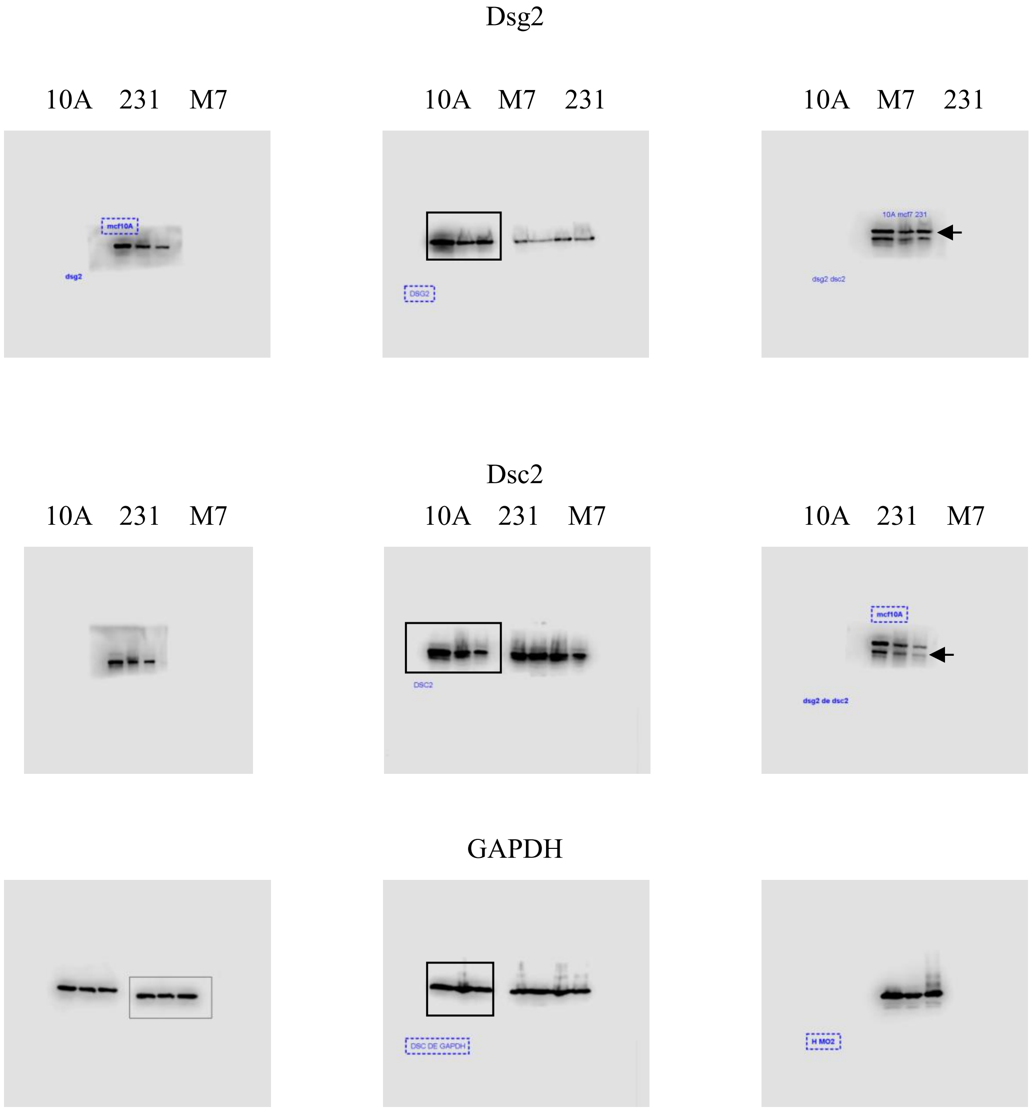

Fig. 3A The comparison of protein levels from four clones (#5, #6, #7 and#8) of shDsg2 and three clones (#31, #32 and#33) of shDsc2 with their controls (shControl1 and shControl2) in MDA-MB-231 cells and MCF-7 cells by western blot, respectively.

Dsg2 in 231

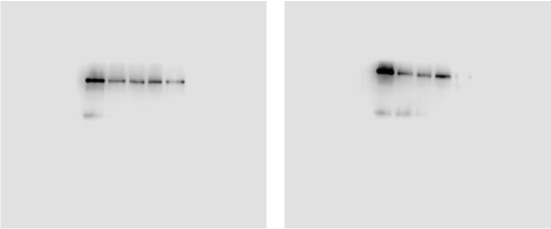

GAPDH in 231

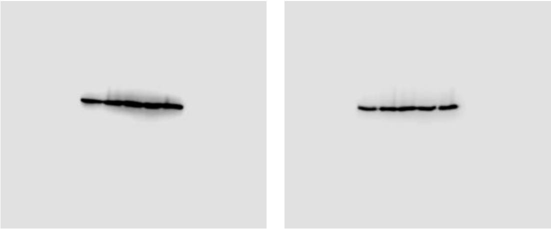

Dsc2 in 231

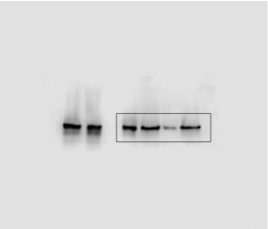

GAPDH in 231

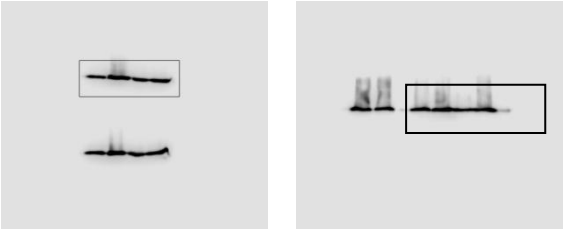

Dsg2 in MCF-7

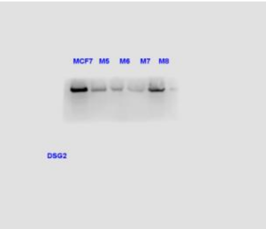

GAPDH in MCF-7

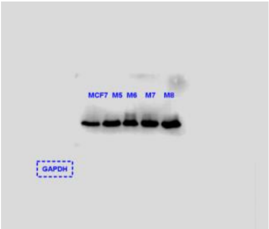

Dsc2 in MCF-7

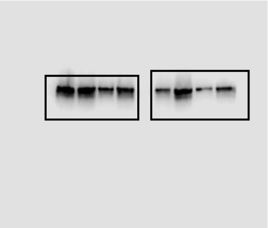

GAPDH in MCF-7

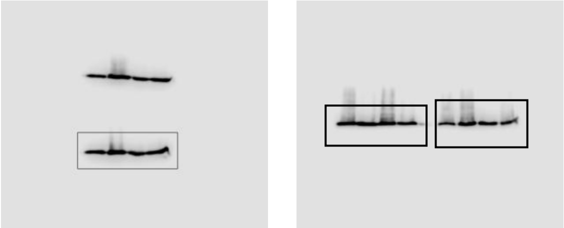

Fig. 6A The expressions of function-associated proteins were determined in shDsg2 and shDsc2 BC cells by western blot

Dsg2 in 231

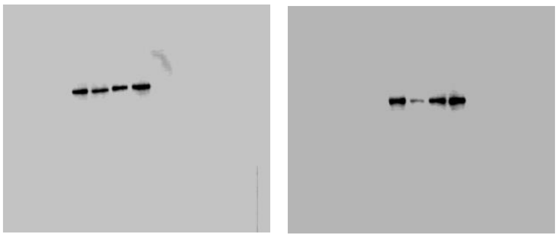

Dsg2 in MCF-7

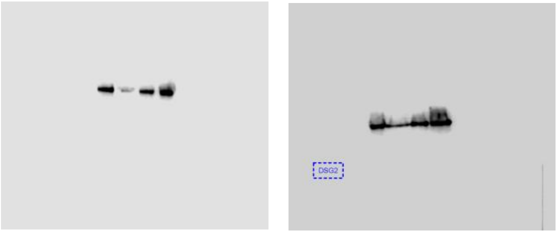

Dsc2 in 231

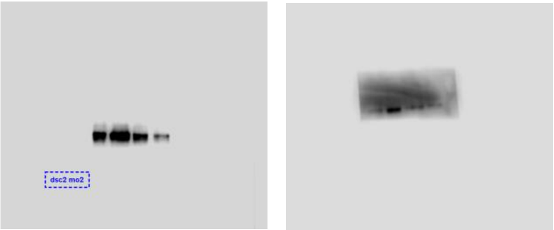

Dsc2 in MCF-7

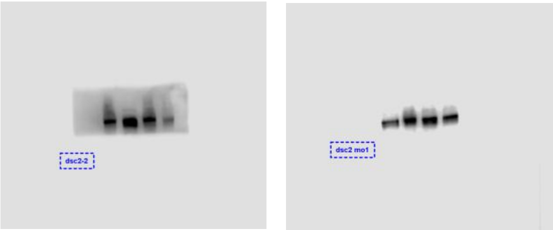

N-cadherin in 231

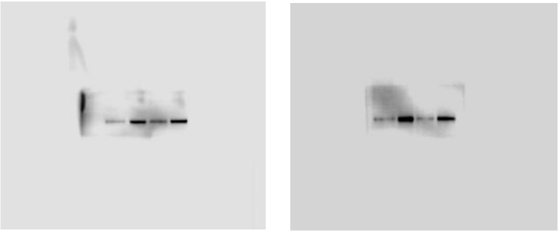

E-cadherin in MCF-7

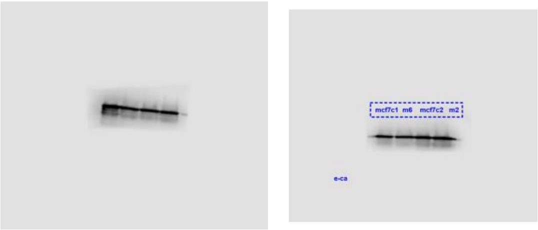

CD133 in 231

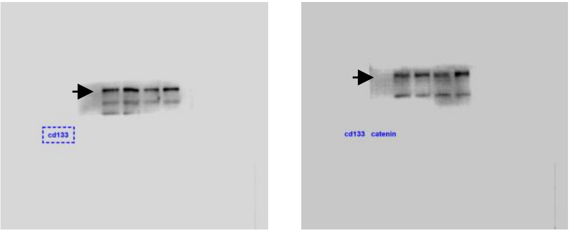

CD133 in MCF-7

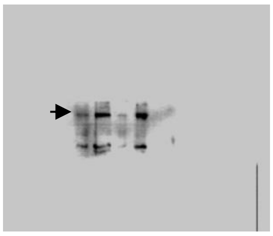

cyclin D1 in 231

cyclin D1 in MCF-7

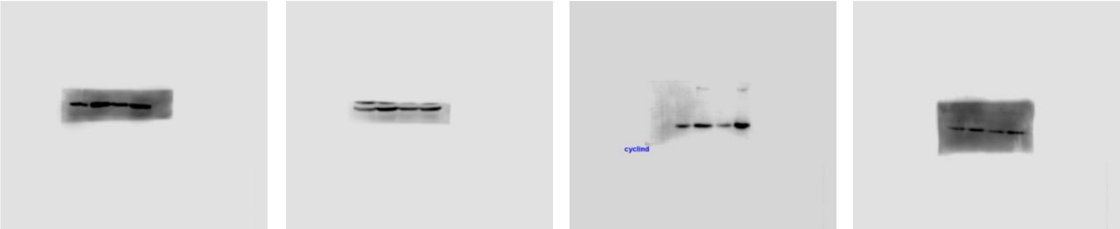

β-catenin in 231

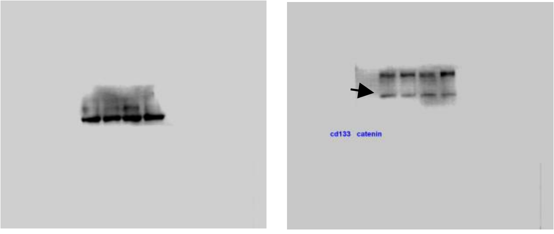

β-catenin in MCF-7

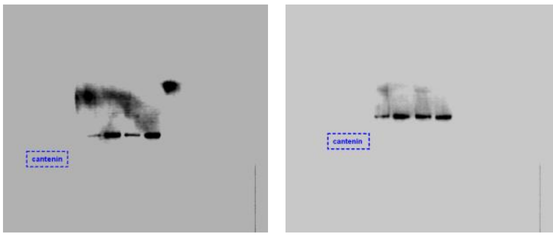

GAPDH in 231

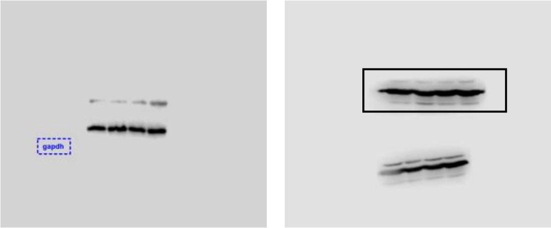

GAPDH in MCF-7

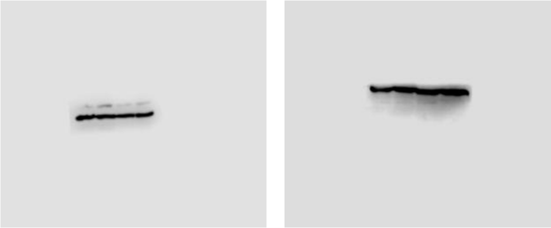

Fig. 6B The expressions of pathway-associated proteins were determined in shDsg2 and shDsc2 BC cells by western blot

Dsg2 in 231

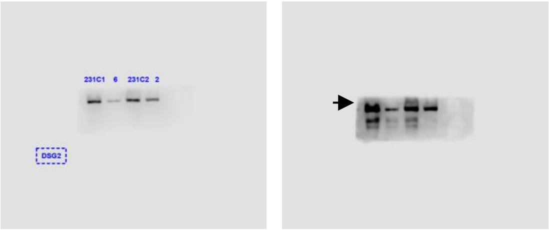

Dsg2 in MCF-7

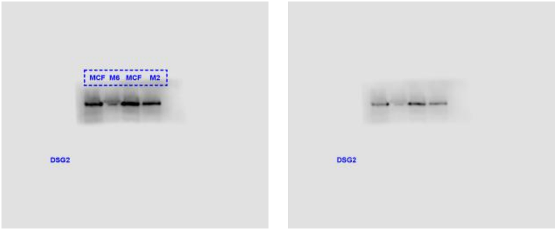

Dsc2 in 231

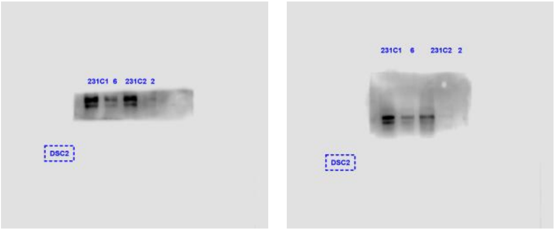

Dsc2 in MCF-7

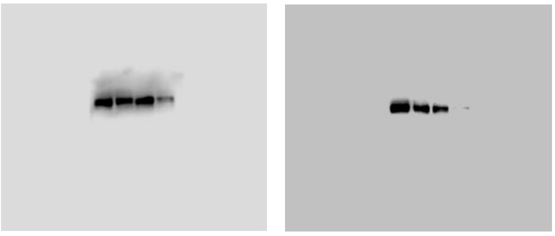

EGFR in 231

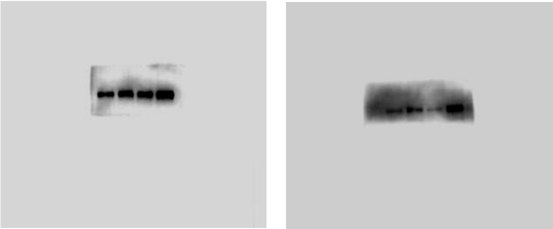

EGFR in MCF-7

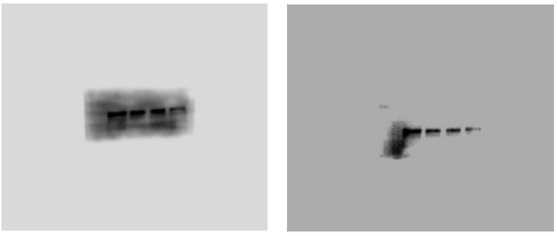

p-EGFR(Y845) in 231

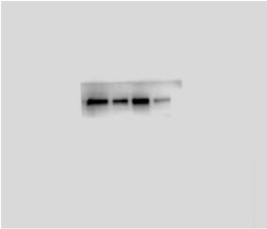

p-EGFR(Y845) in MCF-7

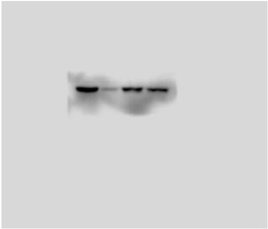

p-EGFR(Y1092) in 231

p-EGFR(Y1092) in MCF-7

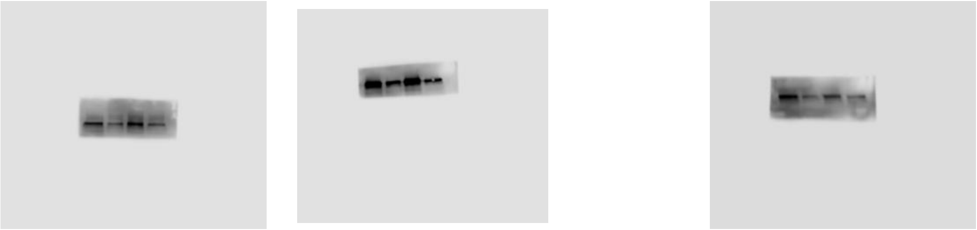

p-AKT(S473) in 231

p-AKT(S473) in MCF-7

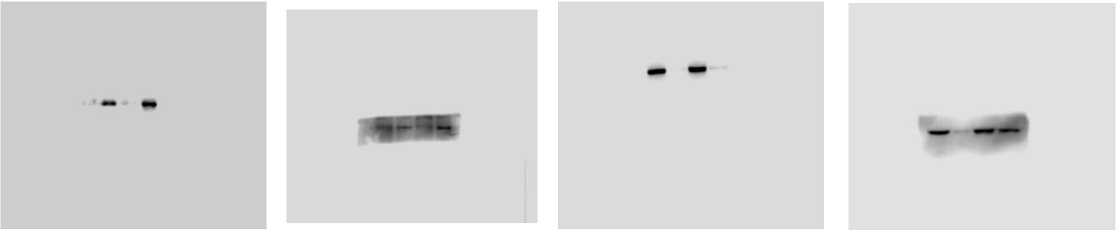

p-ERK(T202/Y204) in 231

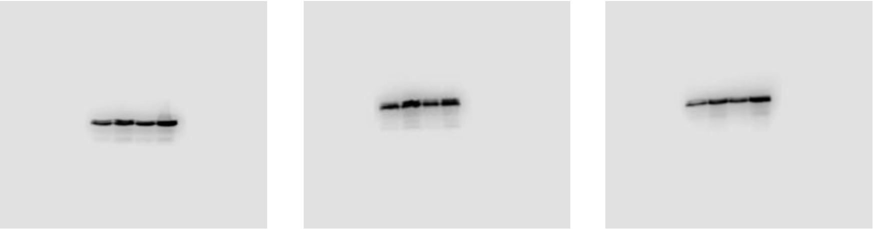

p-ERK(T202/Y204) in MCF-7

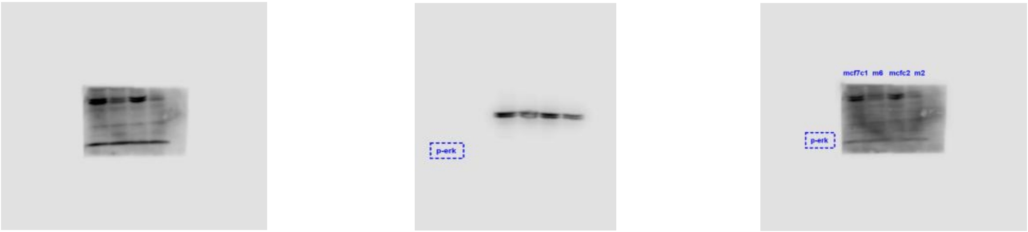

GAPDH in 231

GAPDH in MCF-7

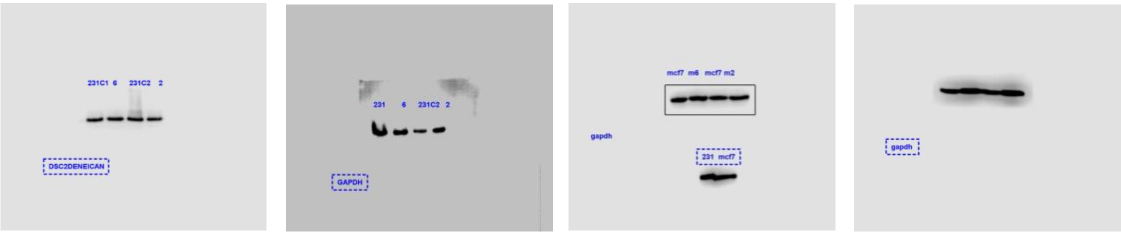

Fig. 7C Western blot analysis of p-AKT and p-ERK expression in response to MK2206 and PD98059 treatment in shDsg2 and shDsc2 MDA-MB-231 cells.

Dsg2 in shDsg2 231 cells

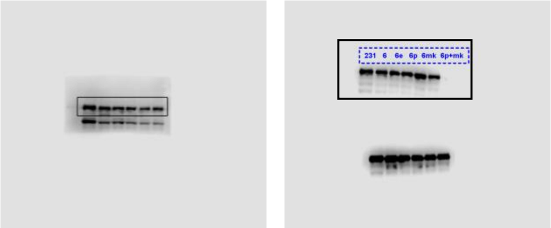

Dsg2 in shDsc2 231 cells

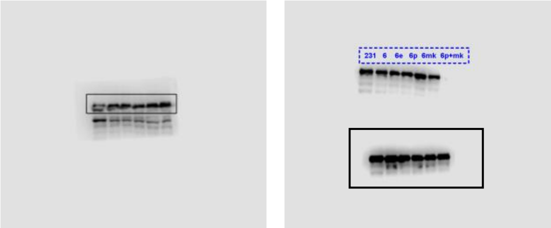

Dsc2 in shDsg2 231 cells

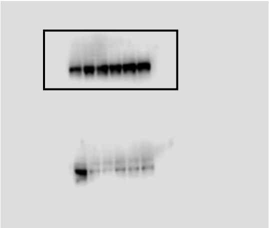

Dsc2 in shDsc2 231 cells

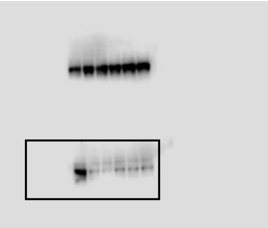

p-ERK in shDsg2 231 cells

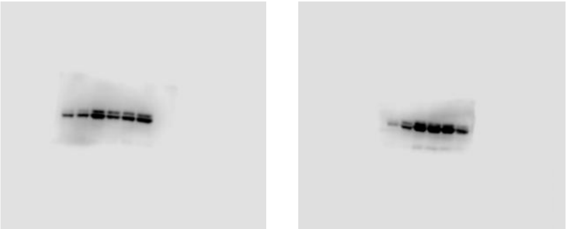

p-ERK in shDsc2 231 cells

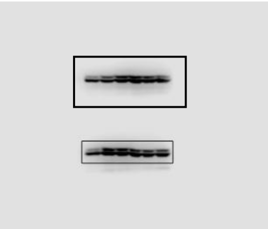

p-AKT in shDsg2 231 cells

p-AKT in shDsc2 231 cells

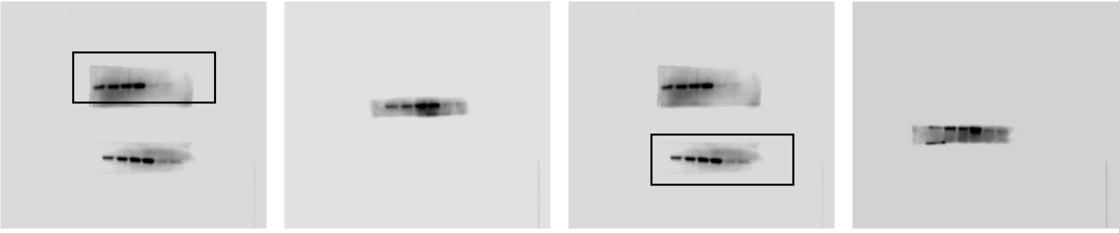

GAPDH in shDsg2 231 cells

GAPDH in shDsc2 231 cells

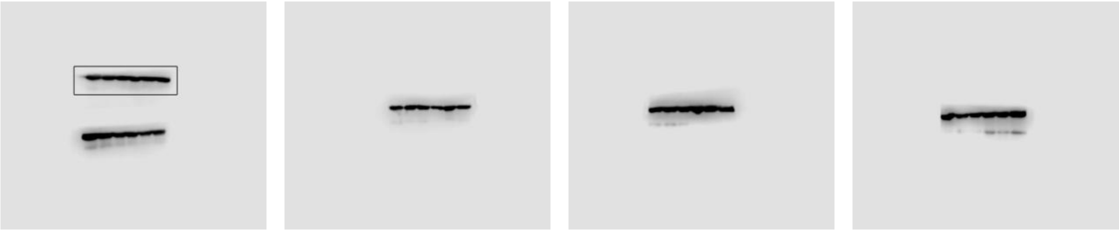

Supplement: Supplementary file 1 — Supplementary Material 1 [file 12885_2024_12229_MOESM1_ESM.pdf]
